# Effects of online mindfulness-based interventions (MBIs) on anxiety symptoms in adults: a systematic review and meta-analysis

**DOI:** 10.1186/s12906-023-04102-9

**Published:** 2023-07-28

**Authors:** Chuntana Reangsing, Pimkanabhon Trakooltorwong, Kunnara Maneekunwong, Jintana Thepsaw, Sarah Oerther

**Affiliations:** 1 School of Nursing, Mae Fah Laung University, Chiangrai, Thailand; 2 Trudy Busch Valentine, School of Nursing, Saint Louis University, Missouri, MO USA

**Keywords:** Anxiety, Adults, Mindfulness, Meta-analysis

## Abstract

**Background:**

An increasing number of studies have documented the effectiveness on various types of face-to-face and online mindfulness-based interventions (MBIs) in reducing anxiety among general population, but there is a scarcity of systematic reviews evaluating evidence of online MBIs on anxiety in adults. Therefore, we examined the effects of online mindfulness-based interventions (MBIs) on anxiety symptoms in adults and explored the moderating effects of participant, methods, and intervention characteristics.

**Methods:**

We systematically searched nine databases through May 2022 without date restrictions. Inclusion criteria were primary studies evaluating online mindfulness-based interventions with adults with anxiety measured as an outcome, a comparison group, and written in English. We used random-effects model to compute effect sizes (ESs) using Hedges’ *g*, a forest plot, and *Q* and *I*^*2*^ statistics as measures of heterogeneity; we also examined moderator analyses.

**Results:**

Twenty-six primary studies included 3,246 participants (39.9 ± 12.9 years old). Overall, online mindfulness-based interventions showed significantly improved anxiety (*g* = 0.35, 95%CI 0.09, 0.62, *I*^*2*^ = 92%) compared to controls. With regards to moderators, researchers reported higher attrition, they reported less beneficial effects on anxiety symptoms (*β*=-0.001, Q_*model*_=4.59, p = .032). No other quality indicators moderated the effects of online mindfulness-based interventions on anxiety.

**Conclusion:**

Online mindfulness-based interventions improved anxiety symptoms in adult population. Thus, it might be used as adjunctive or alternative complementary treatment for adults. However, our findings must be interpreted with caution due to the low and unclear power of the sample in primary studies; hence, high-quality studies are needed to confirm our findings.

**Supplementary Information:**

The online version contains supplementary material available at 10.1186/s12906-023-04102-9.

## Introduction

Anxiety disorders are a common mental health problem [1–3]. Anxiety disorders are characterized by excessive worry that is difficult to control and can be accompanied by physical symptoms including restlessness, being easily fatigued, difficulty concentrating, irritability, or sleep disturbances [2, 4]. Women were more likely to experience mild, moderate, or severe symptoms of anxiety than men [1, 5].

The prevalence of anxiety has increased worldwide. Globally, 45.8 million incident cases of anxiety disorders, 301.4 million prevalent cases and 28.7 million DALYs were estimated in 2019 [3]. Examples, in each country, over 12% of Thai adults have anxiety symptoms [6, 7]. In the United Kingdom (UK), the incidence of anxiety symptoms in young adults rose from 6.2/1000 person-years at risk (PYAK) in 2003 to 15.3/1000 PYAR in 2018 [8]. Terlizzi and Villarro [5] found that around 15% of adults in the United States experienced symptoms of anxiety. In China, approximately 35% of adults experienced with anxiety symptoms [9, 10].

Anxiety disorders can have wide-ranging negative effects on adults’ functioning. They are associated with lower cognitive performance [11] and sleep disturbance [12], and a high risk of somatic illness such as pain or fatigue [13]. Additionally, anxiety is related to chronic disease such as GI diseases [14, 15] and heart disease [13]. Moreover, having anxiety disorder was associated with a low quality of life [16], and a lot of limitation in daily living such as social restriction [17]. Importantly, not only anxiety disorder associated with individuals functioning but also impact to economic burden [18–20]. Anxiety disorder was associated with considerable economic costs owing to lost work productivity and high medical resource use [20, 21]. As a systematic review and meta-analysis by Konnopka and König [22] found that an average of direct cost of anxiety disorder corresponded to 2.08% of health care costs and 0.22% of gross domestic product (GDP), whereas indirect cost, on average, corresponded to 0.23% of GDP.

Pharmacologic treatments for anxiety, such as anxiolytics and anti-depressants, have been effective for helping control symptoms of anxiety in adults, but many are not recommended for long-term use. For instance, benzodiazepine and serotonin reuptake inhibitors (SSRIs) are the drugs of choice for the treatment of anxiety. However, chronic use of benzodiazepine can lead to addiction, and abrupt discontinuation of treatment can lead to withdrawal syndrome [23, 24]. The chronic use of SSRIs can produce side effects such as nervousness, tremors, sweating, nausea, diarrhea, and difficulty falling asleep or frequent awakening [25].

Non-pharmacologic treatments such as cognitive behavioral therapy (CBT) have been used to treat symptoms of anxiety, but once CBT is discontinued, many patients with anxiety become unresponsive or continue to have residual symptoms [26]. Additionally, there are several barriers to CBT delivery, such as insufficient therapists [27]; stigmatization; long waiting times for treatment; and high costs [28, 29]. Thus, alternative and complementary therapies to improve anxiety symptoms are growing. One of these therapies is mindfulness-based intervention (MBIs).

Mindfulness, is a process that leads to a mental state defined by nonjudgmental awareness of one’s experiences, thoughts, physiological states, consciousness, and environment, while fostering openness, curiosity, and acceptance [30, 31]. Thus, mindfulness-based intervention (MBIs) is a practice that allows for self-regulation of the body and mind through body scan, sitting meditation and mindfulness movement such as yoga or other mindfulness exercise [31]. Notable, mindfulness training is recognized as cognitive training because individuals are encouraged to understand the relationship between their thoughts, emotions, and behaviors related anxiety. With this practice, individuals become more aware and can self-regulate their thoughts, emotions, and behaviors related to anxiety [32]. Mindfulness principles are applied to help individuals in identifying an alternative in mood without immediately evaluating or responding to it. This increased internal awareness is then combined with cognitive therapy techniques which teach individuals to disengage from maladaptive patterns of repetitive thoughts that are associated with anxiety symptoms [30]. Researchers have shown that using MBIs to treat adults with symptoms of anxiety has fewer barries when compared to other non-pharmacologic treatments and is cost effective [33]. MBIs refer to a range of therapeutic approaches that guide individuals to use mindfulness techniques, including formal and informal exercises [31, 34], and emphasizes a non-judgmental focus on and awareness of the present moment [31]. Formal exercises that facilitate mindfulness include sitting meditation, mindful movement, and body scanning. Informal exercises include mindful eating and are designed to promote mindful awareness in daily activities [34, 35]. Traditionally, MBIs included a range of formal, daily home-based mindfulness practices informed by mindfulness-based stress reduction (MBSR); mindfulness-based cognitive therapy (MBCT); and adapted mindfulness-based interventions (adapted MBIs). With adapted MBIs, researchers adapted structured sessions of mindfulness-based interventions to be shorter than MBSR and MBCT.

Researchers have conducted meta-analyses on various types of face-to-face and online MBIs to improve anxiety symptoms in the specific population [36–39]. For instance, Lin, Lin [40] found that MBIs significantly improved anxiety in cancer patients (SMD=-3.48, 95%CI-4.07, -2.88, s = 10). Similarly, Li, Sun [41] found MBIs could significantly improve anxiety in nursing students (SMD=-0.45, 95%CI, − 0.73, − 0.17, *p* = .001). In addition, Spijkerman and Bohlmeijer [42] found that online MBIs had a small effect on anxiety (SMD = 0.22, 95%CI.05, 0.39, s = 10). Moreover, Witarto et al. [43] found that online MBIs could improve the severity level of anxiety in adults during the COVID-19 pandemic (g=-0.25, 95%CI, − 0.43, 0.06, p = .008, s = 8). Furthermore, Gong et al. [44] found that online MBIs had a positive impact to reduce anxiety symptoms in university students (SMD=-0.34, 95%CI, − 0.57, − 0.11, p = .004, s = 6). However, all research teams [41–44] included a small number of primary studies (*s* = 5–10), did not specifically included in general adults [40, 44] and did not examine the subgroup analysis to explore the source of heterogeneity [40, 41]. Conducting meta-analysis with a small number of primary studies may overestimate the effect sizes [45, 46].

Importantly, no prior researchers specifically conducted meta-analyses that address the effects of online MBIs on anxiety symptoms and explore the subgroup analysis in the general adult population. Therefore, the purpose of this study was to examine the effects of online MBIs on anxiety symptoms in adult populations. We also explored the moderator effects of source, participants, methods, and intervention characteristics. We hypothesized that adults with anxiety who engaged in online MBIs would have fewer anxiety symptoms than adults who did not engage in online MBIs.

## Methods

### Design

The Preferred Reporting Items of Systematic Reviews and Meta-Analysis (PRISMA) framework guided this study by assisting in the identification, selection, and critical appraisal of the literature [47]. A study protocol was registered at the International Prospective Register of Systematic Reviews, PROSPERO (CRD 42,022,312,239).

### Search strategy and selection criteria

A total of nine electronic databases (i.e., CINAHL with full text, PsycINFO, Ovid Medline, PubMed, Scopus, Cochrane, ProQuest & Theses, Mindfulness Journal, and ScienceDirect) were searched using key terms to capture mobile health or digital interventions, anxiety, and mindfulness-based interventions among adults to retrieve all relevant articles from 2014 to 2022 (See Supplementary Table 1). Subject headings were used in databases when appropriate. The title and abstract of each article were determined independently by the research team for all of the identified articles. Conflicts were resolved by consensus with the senior researcher. Reference lists of the included articles, reviews, and meta-analysis were inspected for additional articles.

### Inclusion and exclusion criteria

The following criteria were used to select relevant studies for inclusion in this systematic review and meta-analyses: (1) studies that included adults with anxiety; (2) studies that used an experimental design (RCT, quasi); (3) the treatment group received MBIs including MBSR, MBCT, and adapted MBIs either with or without guided meditation; (4) the MBIs were administered via the internet or a computer application including virtual classrooms; (5) the control group received a usual (TAU) control group, waitlist control group; (6) the treatment outcome was quantitative anxiety; and (7) studies were written in English. The exclusion criteria were: (1) interventions were just a psychoeducation program and did not involve mind-body exercises for enhancing mindfulness; (2) studies in which the researchers combined MBIs and other forms of therapy (e.g., cognitive behavioral therapy, supportive therapy, antidepressant treatment, or therapies such as yoga, tai-chi, transcendental meditation, acceptance and commitment therapy), making it difficult to distinguish the effects of online MBIs from other therapies because we were specifically interested in the effect of online MBIs on anxiety.

### Study selection and eligibility

Three of the authors (CR, KM, SO) independently assessed the eligibility of all studies that examined the effectiveness of online mindfulness-based interventions on anxiety, based on the selection criteria. Studies involving other groups of participants, such as adolescents and older adults, were excluded. Disagreements between evaluators were resolved by discussion.

### Data extraction and coding

A codebook was developed based on the previous studies [48, 49] to extract data from the eligible studies and revised it during pilot testing with three primary studies. These included five categories [48, 49], which were: source of information, methods, interventions, participants, and outcomes. Source of information included the eligibility criteria and the author, year, funding, country, and publication status. Methods variables included setting, type of comparison group, sampling, and quality indicators such as group assignment, concealed allocation, data collectors masking, intention to treat, fidelity check, power estimation, and group comparison [50]. Interventions variables included the type (MBSR, MBCT, adapted MBIs); format (i.e., mobile application, website-based intervention); whether the intervention was guided, body scan, psychoeducation, group discussion, sitting meditation, or body movement; and whether there was counseling and home assignments. We also extracted days across the intervention, number of weeks in which the intervention was administered, number of intervention sessions, and minutes per session. Participants variables included the total number of participants, their mean age and standard deviation, participants in the intervention group, participants in the control group, number of participants at analysis in both groups, number of dropouts, number of females, number of participants across races, and presence of mood disorders, stress, learning disorders, and the use of drugs. Finally, the outcome variables included anxiety instruments, reliability of scale, mean and standardized anxiety scores, and the effect direction [48, 49].

Data was extracted by two of the authors (PT & JT). Any inconsistencies in data extraction were resolved via discussion between the research team (PT, KM, JT, & SO) and through consultation with the third researcher (CR).

### Statistical analysis

We used SPSS to conduct descriptive statistics for the study characteristics. Comprehensive Meta-Analysis (CMA) was used to compute the effect size (ES) by using the standard mean differences between online MBIs and comparison groups’ posttest anxiety scores [46]. Because the included studies differ in ways that cannot be measured such as intervention delivery, setting features, and more, we assumed that the included studies had different underlying true effect sizes. Therefore, the random-effects model was used because we assumed that the true effect sizes were normally distributed [46]. We also used Hedge’s with 95% confidence interval (CIs) to estimate the ES because it can correct the bias from small study samples [46].

### Heterogeneity assessment

To test the heterogeneity across studies, we used the forest plot, which visually demonstrates the degree to which data from multiple studies overlap with one another. Also, Q statistic was used for exploring the total dispersion; significance indicates heterogeneity [46]. Additionally, we used the *I*^2^ statistic, which is the ratio of effect size variability to total variability indicating the observed study effect sizes are more different from each other than what we would expected due to chance alone [46]. The I^2^ statistic reflects the proportion of variance that is true. A value of 25%, 50%, and 75% reflect low, moderate, and high variability [46].

Finally, we examined the subgroup analyses based on the source of information, participants, method, and intervention characteristics to explore the source of heterogeneity [46, 51]. We used a meta-analytic analog of ANOVA for categorical moderators and meta-regression, an analog of regression analysis for continuous moderators [46, 51].

### Assessment of methodological quality

To assess the methodological quality of primary studies, we used the quality indicators [48, 49] as moderators and examined the difference in effect sizes for studies with and without the quality indicators [46]. For this meta-analysis, quality indicators of methodological strength included concealed allocation, random assignment, data collector blinded, *a priori* power analysis, power analysis completed, comparison of demographic groups, and intention-to-treat analysis [46]. These indicators were analyzed as dichotomous moderators, while attrition was analyzed as a continuous moderator [46, 48, 49].

### Risk of publication bias

To estimate the publication bias, we used the funnel plot, Begg and Mazumdar rank correlation test, and Egger’s bias value [46, 51]. A visually symmetrical funnel-shaped distribution represents the absence of publication bias. The Begg and Mazumdar test computes the rank order correlation (Kendall’s τ) between the standard treatment effect and the variance (standard error, which is primarily affected by the sample size). Significant results suggest that publication bias exists. Similarly, a significant result from the Egger regression test suggests publication bias [46].

## Results

### Demographics of the study

**Fig. 1:**
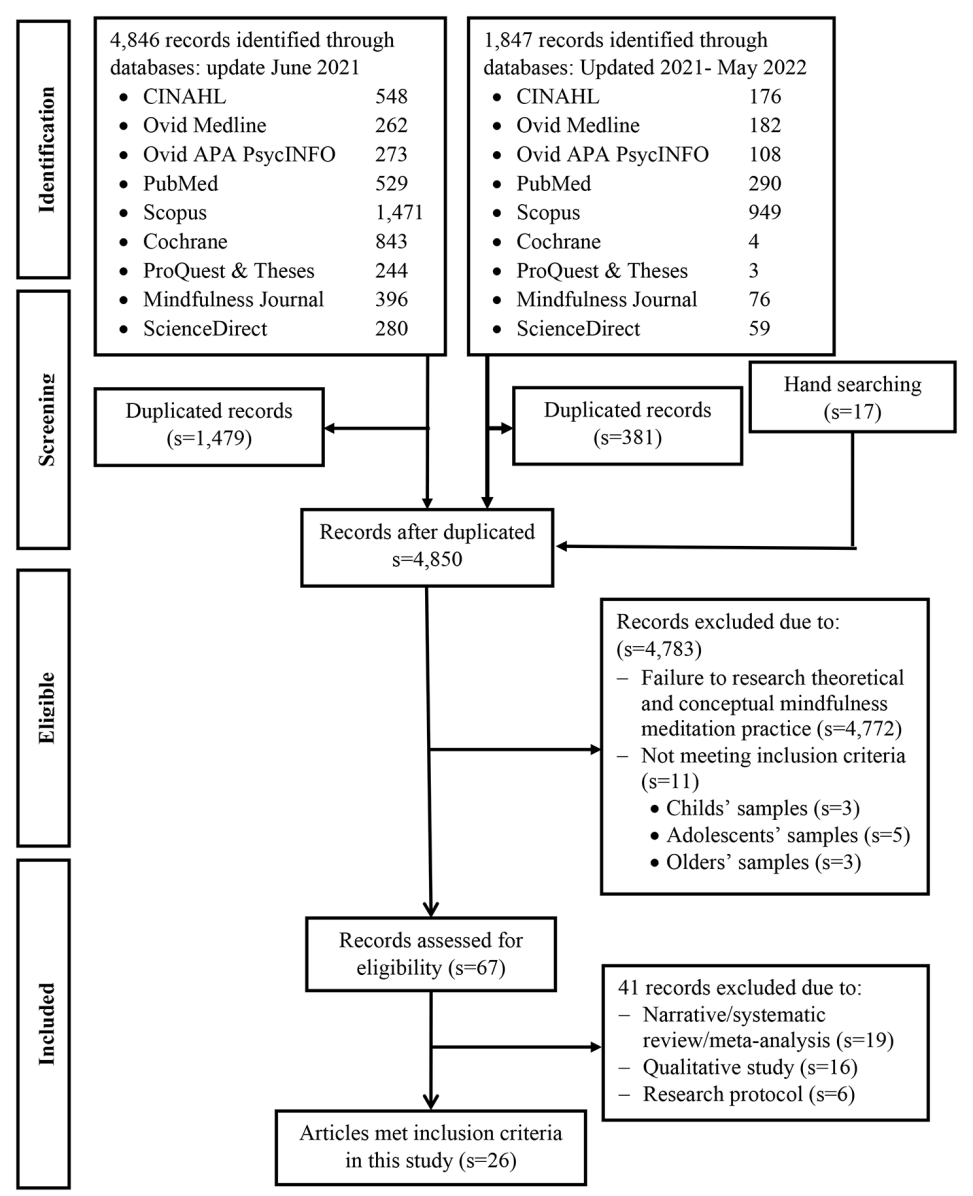
PRISMA Flow of Included Primary Studies

Initial database searches resulted in 4,846 studies in June 2021, and updated search results added 1,847 studies in May 2022. After 1,860 duplicates were removed, 4,833 remained. We found 17 studies through hand ancestry searches. During the review of title and abstract, an additional 4,783 were excluded because they did not include online MBIs and/or any number of inclusion criteria. Of the remaining 67, 41 primary studies were excluded; 19 were narrative/systematic review/meta-analysis; 16 were qualitative studies, and six studies were research protocol without results. Finally, 26 primary studies (S = 26) met inclusion criteria and were included in this systematic review and meta-analysis (See Fig. 1).

The 26 primary studies that met inclusion criteria provided 32 between-group comparisons (K = 32) because some studies had three comparison groups. For example, researchers included three groups, such as the full mindfulness virtual community program (F-MVC); partial MVC; and waitlist control group [52]. We compared groups that were similar except for the online MBI. All 26 primary studies had been published between 2014 and 2022. A total of 3,246 participants were included across the 26 primary studies; 1,979 participants practiced in the online MBIs, and 1,567 participants served as controls. Five of the 26 primary studies were conducted in the United States of America [53–57] as well as five in the United Kingdom [58–62]; three each in Canada [52, 63, 64], Italy [65–67], and China [68–70]; and one each in New Zealand [71], Australia [72], Spain [73], Germany [74], Malaysia [75], Denmark [76], and Japan [77] (See Supplementary Tables 2 and 3). Participants’ mean ages ranged from 20.1 to 63.1 years (See Table 1). Nine instruments were used to determine anxiety in adults including Generalized Anxiety Assessment, GAD-7 (s = 8); Hospital Anxiety and Depression Scale, HADS (s = 7); The Beck Anxiety Inventory, BAI (s = 3); Depression Anxiety Stress Scales, DASS-Anxiety (s = 3); the Patient Health Questionnaire, PHQ (s = 1); the State-Trait Anxiety Inventory, STAI (s = 2); the Patient Reported Outcome Measurement Information System, PROMIS-anxiety (s = 1); and the Brief Symptoms Inventory, BSI-18 (s = 1). Higher scores reflect higher levels of anxiety symptoms (See Table 1) for intervention descriptions including total weeks of interventions, number of sessions/week, and duration of sessions in minutes/session.

**Table 1:** Characteristics of Primary Studies (s = 26)

Characteristics	*s*	Min	Q1	Mdn.	Q3	Max	Mean	SD
Mean age (years)	18	20.1	30.8	39.3	49.4	63.1	39.85	12.9
Total Sample size at analysis								
− MBI group	26	6.0	25.0	38.5	66.0	238.0	52.47	45.8
Control group	26	6.0	22.0	31.0	61.0	260.0	48.98	51.3
Weeks of structured MBI	22	2.0	5.0	8.0	8.0	14.0	7.07	3.3
Days across intervention (length)	22	7.0	28.0	49.0	49.0	91.5	41.89	22.2
Structured MBI session/week	23	0.50	1.0	1.0	7.0	14.0	3.77	3.9
Structured MBI min./session	10	10.0	18.0	35.0	56.3	90.0	40.0	29.1
Dose (length x duration)	9	280.0	609.0	980.0	2712.0	4410.0	1607.0	1421.5
Days after intervention measured	26	0.0	0.0	0.0	91.5	183.0	47.98	70.7
% Attrition, MBI group	25	0.0	7.9	15.0	37.5	79.4	23.06	18.4
% Attrition, Control group	25	0.0	5.0	15.4	26.2	45.5	16.93	13.9

*s* = number of studies providing data, Min = minimum, Q1 = first quartile, Mdn = median, Q3 = third quartile, Max = maximum, MBI = mindfulness-based intervention

### Effects of online mindfulness-based interventions

**Fig. 2:**
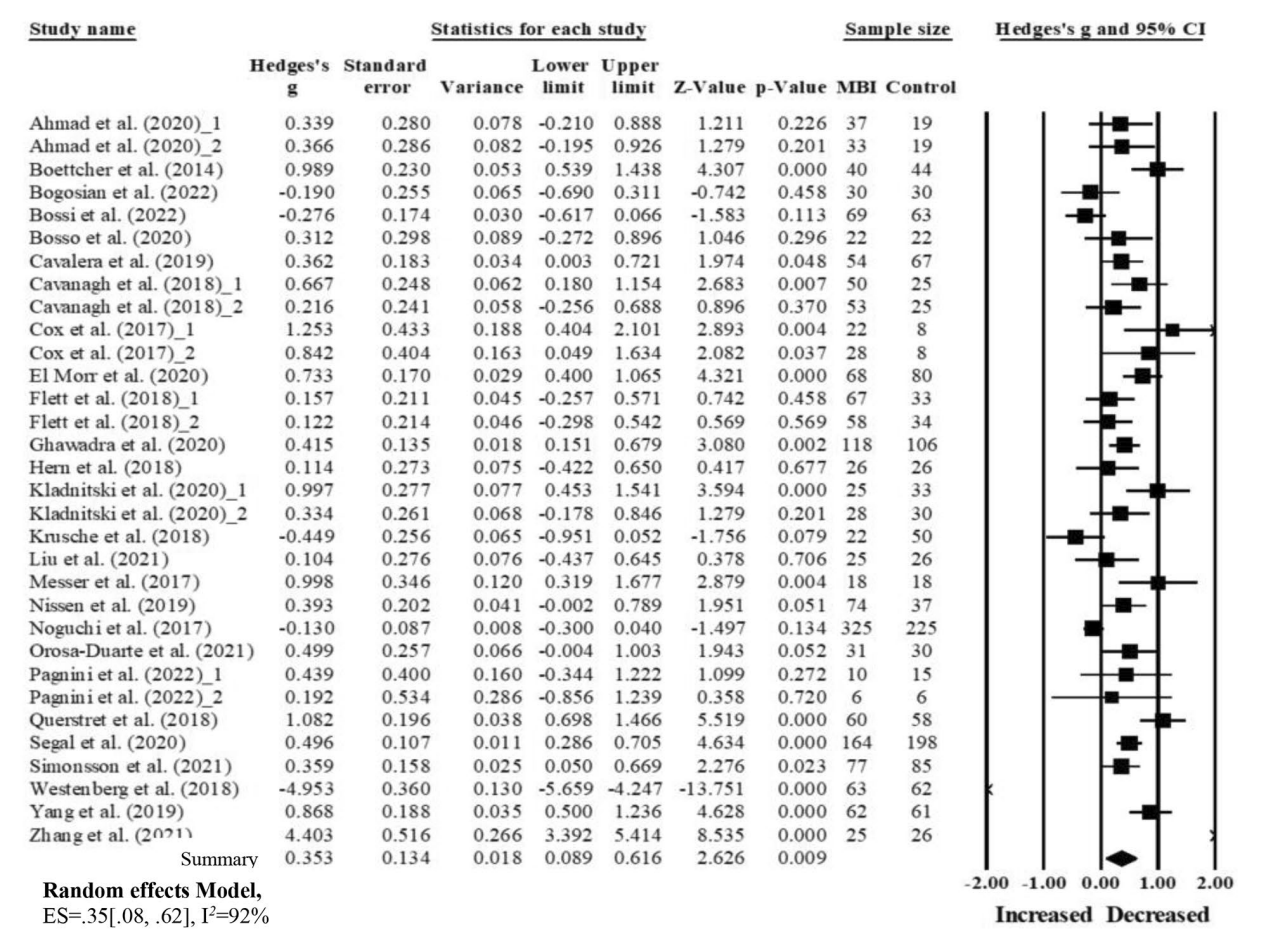
Forrrest plot of the effect of online MBIs on anxiety compared to control groups

Overall, the summary effect size across the 32 comparisons was *g* = 0.35 (95%CI = 0.09, 0.62, *p* = .009, *I*^*2*^ = 92%), indicating that online MBIs had a moderate effect in reducing anxiety symptoms among adults. Of all 32 comparisons, fifteen comparisons had significant positive effects improvement (See Fig. 2).

The online MBIs group’s pre-post comparisons demonstrated significant reduction in anxiety symptoms with an effect size of g = 0.71 (p < .001) for correlated groups (r = .8) and g = 0.67 (p < .001) for uncorrelated groups. The control group’s pre-post effect sizes showed no improvement in anxiety symptoms for the uncorrelated group (g = 0.15, r = .0) and improvement of anxiety for the correlated group (g = 0.21, r = .8) (See Table 2).

**Table 2:** Effect size of online MBI vs. Control groups

Comparison	MM group							
	*k*	ES	*p*(ES)	95% CI	SE	*I* ^*2*^	Q	*p*(Q)
Online MBI vs. Control groups	32	0.353	0.009	0.089, 0.616	0.134	92.1	391.1	< 0.001
Single Online MBI group								
pre- vs. post (r = .0)	26	0.674	< 0.001	0.479, 0.870	0.100	79.6	122.7	< 0.001
pre- vs. post (r = .8)	26	0.714	< 0.001	0.534, 0.893	0.092	95.6	578.4	< 0.001
Single Control group								
pre- vs. post (r = .0)	25	0.146	0.064	− 0.009, 0.301	0.079	58.9	58.5	< 0.001
pre- vs. post (r = .8)	25	0.206	0.006	0.059, 0.353	0.075	91.6	286.7	< 0.001

### Subgroup analyses

Significant heterogeneity existed across the studies (*I*^*2*^ = 92%, Q = 391.1, *p* < .001), indicating that the moderator analysis was warranted. Only one variable had a significant moderator (See Tables 3 and 4) depicting the subgroup analyses. When researchers reported higher attrition, they reported lower reduction in anxiety symptoms (*β*=-0.001, *Q*_*model=*_4.59, *p* = .032). No other quality indicator affected the ES of study.

**Table 3:** Categorical Moderator Results for Depression Comparing MBI versus Control Groups

Moderator	*k*	ES	SE	Var.	95%CI	Z	*p*(Z)	Q_bet_	*p(Q*_*bet*_ *)*	
Source characteristics										
Funding								1.138	0.286	
Unfunded	8	0.535	0.149	0.022	0.243, 0.828	3.588	< 0.001			
Funded	22	0.350	0.090	0.008	0.174, 0.526	3.895	< 0.001			
**Method characteristics**										
Blinded data collection								0.249	0.618	
No	13	0.355	0.117	0.014	0.125, 0.584	3.024	0.002			
Yes	17	0.432	0.101	0.010	0.234, 0.630	4.269	< 0.001			
Intention-to-treat								0.022	0.881	
No	23	0.393	0.090	0.008	0.216, 0.570	4.351	< 0.001			
Yes	7	0.420	0.159	0.025	0.108, 0.733	2.637	0.008			
Concealed allocation								1.126	0.289	
No	10	0.285	0.134	0.034	0.022, 0.549	2.121	0.034			
Yes	20	0.462	0.099	< 0.001	0.269, 0.655	4.685	< 0.001			
Baseline characteristics equal across groups								0.689		0.406
No	6	0.284	0.191	0.037	− 0.091, 0.658	1.484	0.138			
Yes	14	0.477	0.132	0.017	0.217, 0.736	3.604	< 0.001			
Power of sample								0.463	0.496	
No	10	0.232	0.134	0.018	− 0.031, 0.496	1.728	0.084			
Yes	8	0.364	0.139	0.019	0.091, 0.637	2.611	0.009			
Fidelity								0.037	0.847	
No	29	0.402	0.080	0.006	0.246, 0.559	5.032	< 0.001			
Yes	1	0.312	0.463	0.214	− 0.595, 1.219	0.673	0.501			
**Intervention characteristics**										
MBI type								1.561	0.458	
MBSR	3	0.299	0.243	0.059	− 0.178, 0.776	1.229	0.219			
MBCT	2	0.740	0.292	0.085	0.168, 1.312	2.535	0.011			
Adapted MBI	24	0.383	0.091	0.008	0.206, 0.561	4.226	< 0.001			
Home Assignment								0.210	0.647	
No	20	0.454	0.096	0.009	0.267, 0.642	4.749	< 0.001			
Yes	8	0.371	0.154	0.024	0.070, 0.673	2.413	0.016			
Structure MBI format								1.184	0.553	
Individual	7	0.358	0.168	0.028	0.029, 0.688	2.134	0.003			
Group	21	0.445	0.097	0.009	0.254, 0.636	4.574	< 0.001			
Mixed (Individual + group)	2	0.116	0.299	0.089	− 0.470, 0.701	0.387	0.699			
**Outcome measure**										
Days after intervention measured								0.655		0.418
Immediate post-MBI	18	0.448	0.098	0.010	0.256, 0.640	4.573	< 0.001			
Delayed follow-up	12	0.320	0.124	0.015	0.077, 0.563	2.580	0.010			

*k* = number of comparisons, Q = heterogeneity statistics, SE *=* standard error, MBSR = mindfulness-based stress reduction, MBCT = mindfulness-based cognitive therapy, Adapted MBIs = Adapted mindfulness-based interventions, Var.=variance, NR = not reported

**Table 4:** Continuous Moderators of the Effects of Mindfulness based intervention on Depression

Moderator	*k*	Slope	SE	Tau^2^	Q_model_	*p*
Study characteristic						
Publication year	30	− 0.072	0.04	0.09	3.23	0.072
**Sample characteristic**						
Age (mean)	22	− 0.001	0.01	0.10	0.03	0.855
**Method characteristic**						
%Attrition	29	− 0.001	0.01	0.06	4.59	0.032
Reliability of anxiety instruments	12	-2.38	2.67	0.10	0.08	0.375
**Intervention characteristics**						
Intervention length (total week)	26	− 0.011	0.02	0.09	0.20	0.653
Online MBI sessions per week	27	− 0.024	0.02	0.08	1.69	0.194
Duration of Online MBI min./session	9	0.000	0.00	0.05	0.00	0.944
Dose (Length x Duration)	8	0.00	0.00	0.05	0.01	0.943
Days After intervention measured	30	− 0.001	0.00	0.10	0.23	0.635

*k* = number of comparisons, Q = heterogeneity statistics

### Publication Bias

**Fig. 3:**
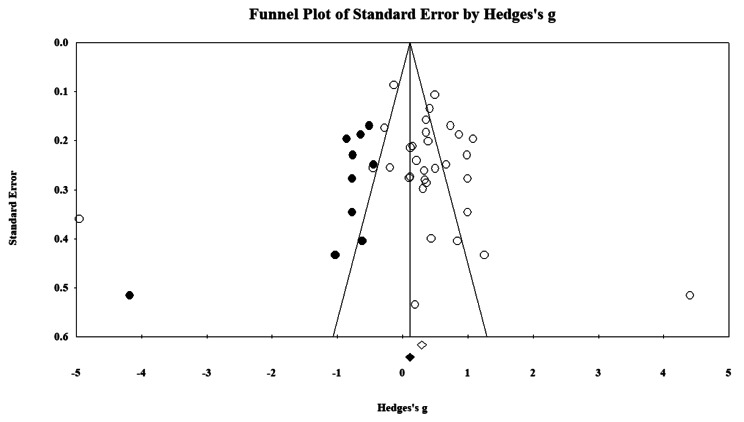
Funnel plot

The funnel plot appeared asymmetrical (See Fig. 3). Egger’s test of the intercept was 0.975 and non-significant (95%CI, -2.16, 4.12, *t* = 0.63, *df* = 30, *p* = .265); Begg and Mazumdar rank correlation test indicated a non-significant Kendall’s tau of 0.01 (*p* = .454), suggesting publication bias was unlikely. However, the power of the tests is low due to a small number of comparisons (K = 32). Thus, the findings should be interpreted with caution.

### Quality of the included studies

After assessing the quality of studies, we revealed that 22 RCTs examined the effectiveness of online MBIs on anxiety in adults. Twenty primary studies provided information on allocation concealment, 17 studies described blinding of outcome assessment, 8 trials addressed the power of sample, and only 1 study reported the fidelity of intervention. Supplemental Table 2 presents the quality of each included study.

## Discussion

This is the first systematic review and meta-analysis exclusively evaluating the effectiveness of online MBIs on anxiety symptoms in adults. Overall, MBIs have a moderate effect (*g* = 0.35) on anxiety symptoms in adults compared to control groups. One possible reason might be that with mindfulness practicing, individual pays more attention at the present moment without judgement [32]. Then, an individual learns how to manage their ruminative thoughts/wondering mind related anxiety [32]. Our finding is different from a previous published meta-analysis [42] assessing the effect of online MBIs on psychological outcomes. This meta-analysis found that MBI was small effective in reducing anxiety symptoms (SMD = 0.22, 95%CI.05, 0.39) [42]. However, they included a small number of primary studies (*s* = 10) which might lead to an overestimate of ES [46] and an inaccurate precision of confidence interval for the common effect size in meta-analysis [78]. Also, these results were different from our study because their meta-analysis included Internet-based mindfulness treatment (*s* = 1), MBSR (*s* = 2), MBCT (*s* = 2) and ACT (*s* = 5). In our study, we only included MBSR, MBCT and adapted MBIs, which are operationalized the mindfulness based on the philosophical perspective of Buddhist teaching using formal meditation as the main interventional component [79]. We did not include ACT because it relies on the Relational Frame Theory, which is derived from a functional contextualism philosophical perspective and focuses on the behavior of individuals within their historical and situational context [79, 80]. Therefore, our meta-analysis is novel in that it provides a comprehensive examination of the effect of online MBI on anxiety in adults with a greater number of primary studies (*s* = 26) than the prior meta-analysis (s = 10, [42]. In addition, we conducted moderator analyses, which provide future research directions.

Although gender difference might be a related factor of anxiety disorder [81, 82], most primary research teams were not report the number of participants in each gender result to a limiting for subgroup analysis to explore how gender affects to the ES. Thus, we recommend the primary researchers address the number of participants based on gender.

Attrition rate is considered a factor affecting the online MBIs’ effect. We found that when the attrition rate increased, the effects of online MBIs was reduced, indicating an increase in anxiety scores. Since a higher attrition also results in a smaller number of participants in the analysis, the precision of the effect size is reduced [46, 83, 84]. We recommend that future researchers account for attrition during recruitment of participants.

### Strengths and limitations

Ours was the first systematic review and meta-analysis of online MBIs on anxiety symptoms in adults. We did a moderator analysis on the biggest number of primary studies (s = 26) to date. Yet, there are certain drawbacks to this meta-analysis. Initially, we limited our search to main research written in English; relevant studies written in other languages would have been missed. Researchers in the future should incorporate papers published in different languages. Second, due to insufficient data reporting, we did not investigate the impact of several key parameters on the effect magnitude. For example, most researchers did not consider intervention fidelity, which was a constraint for investigating this characteristic that influences effect magnitude. Lastly, most investigations examined outcomes shortly after the intervention was completed (s = 19); long term effects were not measured. Thus, more long-term MBIs studies on anxiety symptoms in adults are needed.

### Implications and recommendations

This systematic review and meta-analysis provides evidence for the use of online MBIs in adults with anxiety. Specifically, nurses and health professionals might consider using online MBIs as an adjunctive or alternative complementary treatment to improve anxiety, especially when there are insufficient mental health professionals. Electronic services such as online MBIs might benefit adults who are concerned about negative perceptions of anxiety treatment. Researchers should explore the long-term effects of online MBIs on anxiety in adults. Finally, researchers should account for attrition during the recruitment of participants.

## Conclusion

In conclusion, we found that online MBIs have a moderate effect in decreasing anxiety symptoms in adults. Nurses and mental health professionals may use online MBIs as adjunctive or alternative complementary treatment for managing anxiety symptoms in adults. Also, health providers might engage high-risk adults in online MBIs to prevent anxiety disorders. However, our findings must be interpreted with caution due to the low and unclear power of the sample in primary studies; hence, high-quality studies are needed to confirm our findings.

## Electronic supplementary material

Below is the link to the electronic supplementary material.

**Additional file 1: Supplementary Table 1**. An example of the electronic search strategy. **Supplementary Table 2**. Quality Indicators of Included Primary Studies (s=26). **Supplementary Table 3**. Summary Demographic of Included Primary Studies (s=26)

## Data Availability

The datasets used and/or analyzed during the current study available from the corresponding author on reasonable request.
