# Supplementary material for: Effects of online mindfulness-based interventions (MBIs) on anxiety symptoms in adults: a systematic review and meta-analysis
Source: BMC Complement Med Ther. 2023 Jul 28;23:269. doi: 10.1186/s12906-023-04102-9 (PMC10386675; doi:10.1186/s12906-023-04102-9)
Supplement: Supplementary file 1 — Additional file 1: Supplementary Table 1. An example of the electronic search strategy. Supplementary Table 2. Quality Indicators of Included Primary Studies (s=26). Supplementary Table 3. Summary Demographic of Included Primary Studies (s=26) [file 12906_2023_4102_MOESM1_ESM.docx]

**Supplementary Table**. 1. An example of the electronic search strategy

**PubMed**

| **No.** | **Searches** | **Results** |
| --- | --- | --- |
| 1 | mindful* OR meditat* | 15,645 |
| 2 | (mobile AND (program or intervention)) OR (smartphone app*) OR (web* intervention) OR (online intervention) OR (ehealth OR mHealth or telehealth OR (internet-deliver*) AND (program OR intervention)) | 168,916 |
| 3 | (mobile AND (program or intervention)) OR (smartphone app*) OR (web* intervention) OR (online intervention) OR (ehealth OR mHealth or telehealth OR (internet-deliver*) AND (program OR intervention)) AND mindful* OR meditat* | 808 |
| 4 | (mobile AND (program or intervention)) OR (smartphone app*) OR (web* intervention) OR (online intervention) OR (ehealth OR mHealth or telehealth OR (internet-deliver*) AND (program OR intervention)) AND mindful* OR meditat* Filters: Clinical Trial | 174 |
| 5 | (mobile AND (program or intervention)) OR (smartphone app*) OR (web* intervention) OR (online intervention) OR (ehealth OR mHealth or telehealth OR (internet-deliver*) AND (program OR intervention)) AND mindful* OR meditat* Filters: Clinical Trial, Meta-analysis | 221 |
| 6 | (mobile AND (program or intervention)) OR (smartphone app*) OR (web* intervention) OR (online intervention) OR (ehealth OR mHealth or telehealth OR (internet-deliver*) AND (program OR intervention)) AND mindful* OR meditat* Filters: Clinical Trial, Meta-analysis, Randomized controlled trial | 221 |
| 7 | (mobile AND (program or intervention)) OR (smartphone app*) OR (web* intervention) OR (online intervention) OR (ehealth OR mHealth or telehealth OR (internet-deliver*) AND (program OR intervention)) AND mindful* OR meditat* Filters: Clinical Trial, Meta-analysis, Randomized controlled trial, Review | 351 |
| 8 | (mobile AND (program or intervention)) OR (smartphone app*) OR (web* intervention) OR (online intervention) OR (ehealth OR mHealth or telehealth OR (internet-deliver*) AND (program OR intervention)) AND mindful* OR meditat* Filters: Clinical Trial, Meta-analysis, Randomized controlled trial, Review, Systematic Review | 391 |
| 9 | (mobile AND (program or intervention)) OR (smartphone app*) OR (web* intervention) OR (online intervention) OR (ehealth OR mHealth or telehealth OR (internet-deliver*) AND (program OR intervention)) AND mindful* OR meditat* Filters: Clinical Trial, Meta-analysis, Randomized controlled trial, Review, Systematic Review, English | 385 |

**Supplementary Table 2.** Quality Indicators of Included Primary Studies (*s*=26)

| **Author(s)/Years** | **Assignment into groups** | **Concealed Allocation** | **Masked data collector** | **Intention-to-treat** | **Power of Sample** | **Compared groups**  **at baseline** | **Baseline**  **characteristics equal** | **Fidelity check** |
| --- | --- | --- | --- | --- | --- | --- | --- | --- |
| Ahmad et al. (2020)_1 [35] | 1 | 1 | 1 | 0 | 0 | 0 | NR | 0 |
| Ahmad et al. (2020)_2 [35] | 1 | 1 | 1 | 0 | 0 | 0 | NR | 0 |
| Boettcher et al. (2014) [57] | 1 | 1 | 1 | 0 | NR | 1 | 1 | 0 |
| Bossi et al. (2022) [48] | 2 | 0 | 0 | 0 | 1 | 0 | NR | 0 |
| Bosso et al. (2020) [36] | 2 | 0 | 0 | 1 | 0 | 0 | NR | 1 |
| Cavalera et al. (2019) [49] | 1 | 0 | 0 | 1 | 0 | 1 | 0 | 0 |
| Cavanagh et al. (2018)_1 [42] | 1 | 1 | 1 | 1 | NR | 1 | 1 | 0 |
| Cavanagh et al. (2018)_2 [42] | 1 | 1 | 1 | 1 | NR | 1 | 1 | 0 |
| Cox et al. (2017)_1 [37] | 1 | 1 | 1 | 0 | NR | 0 | NR | 0 |
| Cox et al. (2017)_2 [37] | 1 | 1 | 1 | 0 | NR | 0 | NR | 0 |
| El Morr et al. (2020) [46] | 1 | 1 | 0 | 0 | NR | 1 | 0 | 0 |
| Flett et al. (2018)_1 [54] | 1 | 0 | 1 | 0 | 1 | 1 | 1 | 0 |
| Flett et al. (2018)_2 [54] | 1 | 0 | 1 | 0 | 1 | 1 | 1 | 0 |
| Ghawadra et al. (2020) [58] | 1 | 0 | 0 | 0 | 1 | 1 | 0 | 0 |
| Hern et al. (2018) [62] | 1 | 1 | 1 | 0 | 0 | 0 | NR | 0 |
| Kladnitski et al. (2020)_1 [55] | 1 | 1 | 0 | 0 | 1 | 1 | 1 | 0 |
| Kladnitski et al. (2020)_2 [55] | 1 | 1 | 0 | 0 | 1 | 1 | 1 | 0 |
| Krusche et al. (2018) [44] | 1 | 1 | 1 | 0 | 0 | 1 | 0 | 0 |
| Liu et al. (2021) [51] | 2 | 0 | 0 | 0 | NR | 1 | 0 | 0 |
| Messer et al. (2017) [38] | 1 | 0 | 0 | 0 | 1 | 1 | 1 | 0 |
| Bogosian et al. (2022) [41] | 1 | 1 | 1 | 1 | NR | 1 | 1 | 0 |
| Nissen et al. (2019) [59] | 2 | 0 | 1 | 1 | NR | 1 | 0 | 0 |
| Noguchi et al. (2017) [60] | 1 | 1 | 0 | 0 | 0 | 1 | 1 | 0 |
| Orosa-Duarte et al.(2021) [56] | 1 | 0 | 0 | 0 | NR | 0 | NR | 0 |
| Pagnini et al. (2022)_1 [50] | 1 | 1 | 0 | 0 | 0 | 1 | 1 | 0 |
| Pagnini et al. (2022)_2 [50] | 1 | 1 | 0 | 0 | 0 | 1 | 1 | 0 |
| Querstret et al. (2018) [45] | 1 | 1 | 1 | 1 | NR | 1 | 1 | 0 |
| Segal et al. (2020) [47] | 1 | 1 | 1 | 0 | 1 | 0 | NR | 0 |
| Simonsson et al. (2021) [39] | 1 | 1 | 1 | 0 | NR | 0 | NR | 0 |
| Westernberg et al. (2018) [61] | 1 | 0 | 1 | 0 | NR | 1 | 0 | 0 |
| Yang et al. (2019) [52] | 1 | 1 | 1 | 0 | 0 | 1 | 1 | 0 |
| Zhang et al. (2021) [53] | 1 | 0 | 1 | 0 | NR | 1 | 0 | 0 |

Quality indicators: 0 = No, 1 = Yes, ND = no dropouts; NR = not reported, except, Assignment into groups: 1 = individual randomization, 2 = non-randomization

**Supplementary Table 3.** Summary Demographic of Included Primary Studies (*s*=26)

| # | Author/Funding | Study Design | Samples/Setting | Age mean±SD | MMI/Control | MMIs Characteristics | F/U  (mo.) | Measures | |
| --- | --- | --- | --- | --- | --- | --- | --- | --- | --- |
| 1 | Ahmad et al. [35]  Funded | RCT | Undergraduate students in Toronto  Canada | 25.01  (6.71) | 37/19 | - Web-based mindfulness virtual community intervention; 8 weeks with 12 video-based modules, peer-to peer discussion and brief guided videoconference | 0 | | BAI |
|  | Ahmad et al. [35]  Funded | RCT |  | 24.57  (6.31) | 33/19 | - 8 weeks long with 12 video-based modules | 0 | | BAI |
| 2 | Boettcher et al. [57]  Funded | RCT | Patients with social anxiety disorder, general anxiety disorder  German | 38.57  (10.35) | 40/44 | - internet-based mindfulness intervention including brief, instructive audio files presenting mindfulness exercises (sitting meditation, mindfulness movement) | 0 | | BAI |
| 3 | Bogosian et al. [41]  Funded | RCT | Patients with Parkinson disease  UK | 60.87  (10.1) | 30/30 | - Mindfulness-based group therapy; 8 sessions over 8 weeks via videoconference through Skype (guided mindfulness meditation) | 4.5 | | HADS |
| 4 | Bossi et al. [48]  Funded | Non RCT | Italian adults  Italy | NR | 69/63 | - Online mindfulness training program (guided mindfulness meditation) | 0 | | DASS |
| 5 | Bosso et al. [36]  No funded | Non RCT | College students in South Florida  USA | NR | 22/22 | - Mindfulness application over 5 wks. (10 minutes per day) | 0 | | DASS |
| 6 | Cavalera et al. [49]  Funded | RCT | Patients with multiple sclerosis  Italy | 42.77  (8.73) | 54/67 | - Online mindfulness meditation over 8 wks.; music meditation, discussion and home exercise | 6 | | HADS |
| 7 | Cavanagh et al. [42]  No funded | RCT | University staff and students  UK | 30.81  (11.82) | 50/25 | - Online mindfulness-based intervention; sitting meditation, walking meditation, and guided meditation | 0 | | PHQ |
|  | Cavanagh et al. [42]  No funded | RCT |  | 31.24  (11.65) | 53/25 |  | 0 | | PHQ |
| 8 | Cox et al. [37]  Funded | RCT | Patients in intensive care unit  USA | 49.29  (14.67) | 22/8 | - Telephone—based mindfulness training; guided meditation, discussion | 6 | | GAD-7 |
|  | Cox et al. [37]  Funded | RCT |  | 49.26  (15.44) | 22/8 |  | 6 | | GAD-7 |
| 9 | El Morr et al. [46]  Funded | RCT | University students  Canada | 22.55  (6.1) | 68/80 | - Web-based mindfulness virtual community intervention; over 8 wks. with 12 video-based modules with psychoeducation, peer-to-peer discussion and live videoconference | 0 | | BAI |
| 10 | Flett et al. [54]  Funded | RCT | University students  New Zealand | 20.08  (2.8) | 67/33 | - Mobile mindfulness meditation; Head space including formal meditation practices | 0 | | HADS |
|  | Flett et al. [54]  Funded | RCT |  | 20.08  (2.8) | 58/34 | - Mobile mindfulness meditation; Smiling mind including mindful breathing, body scan, mindful eating, sitting meditation | 0 | | HADS |
| 11 | Ghawadra et al. [58]  No funded | RCT | Ward nurses  Malaysia | NR | 118/106 | - Mindfulness-based training including 2-hr workshop followed by 4 wks. of self-practice guided | 0 | | DASS |
| 12 | Hern et al. [62]  No funded | RCT | People with depression and anxiety following spinal cord injury  UK | 44.52  (10.64) | 26/26 | - 8-weeks online mindfulness intervention; breath awareness, body scanning, kindness and informal meditation | 3 | | HADS |
| 13 | Kladnitski et al. [55]  Funded | RCT | Patients with depression or anxiety disorders  Australia | 39.71  (11.46) | 25/33 | - mindfulness training program; guided meditation | 3 | | GAD-7 |
|  | Kladnitski et al. [55]  Funded | RCT |  | 38.95  (12.41) | 28/30 |  | 3 | | GAD-7 |
| 14 | Krusche et al. [44]  Funded | RCT | Pregnant women  UK | 32.7  (NR) | 22/50 | - Online mindfulness program; formal and informal mindfulness meditation over 8 wks. | 0 | | GAD-7 |
| 15 | Liu et al. [51]  Funded | Non RCT | Patients with fever  China | 34.82  (12.48) | 25/26 | - Brief mindfulness intervention; light music via WeChat, guided meditation | 0 | | GAD-7 |
| 16 | Messer et al. [38]  No funded | RCT | Patients with cancer  USA | NR | 18/18 | - Internet training in mindfulness meditation; guided meditation audio clips | 0 | | HADS |
| 17 | Nissen et al. [59]  Funded | Randomized Cluster | Patients with cancer  Denmark | NR | 74/37 | - Internet-delivered mindfulness-based cognitive therapy; 8-week modules including audio exercise, writing tasks, cancer-specific patient examples and videos | 6 | | HADS |
| 18 | Noguchi et al. [60]  Funded | RCT | Adults with mild depression  Japan | 43.35  (11.3) | 325/225 | - Five-minute internet-based cognitive behavioral therapy | 12 wks. | | GAD-7 |
| 19 | Orosa-Duarte et al. [56]  No funded | RCT | Health care students  Spain | NR | 31/30 | - Mindfulness-based mobile application; 8 sessions over 8 weeks | 0 | | STAI |
| 20 | Pagnini et al. [50]  No funded | RCT | Patients with amyotrophic lateral sclerosis and their caregivers  Italy | 63.10  (8.40) | 10/15 | - Online non-meditative mindfulness intervention via website including video and written contents about mindfulness and two daily exercises | 6 | | HADS |
|  | Pagnini et al. [50]  No funded | RCT |  | 60.00  (10.10) | 6/6 |  | 6 | | HADS |
| 21 | Querstret et al. [45]  No funded | RCT | Adults with non-clinical disease  UK | NR | 60/58 | - Online mindfulness-based cognitive intervention over 8 wks. | 0 | | GAD-7 |
| 22 | Segal et al. [47]  Funded | RCT | Patients with residual depressive symptoms  Canada | 48.30  (14.90) | 164/198 | - Online mindfulness-based cognitive therapy over 8 wks. | 3 | | GAD-7 |
| 23 | Simonsson et al. [39]  Funded | RCT | University students  USA | NR | 77/85 | - Online mindfulness program; over 8 wks. via Zoom | 1 | | PROMIS |
| 24 | Westernberg et al. [61]  No funded | RCT | Upper extremity patients  USA | 54.50  (15.00) | 63/62 | - Brief mindfulness video exercise | 0 | | STAI |
| 25 | Yang et al. [52]  Funded | RCT | Pregnant women  China | 30.85  (4.48) | 62/61 | - Online mindfulness intervention over 8 wks. via WeChat | 0 | | GAD-7 |
| 26 | Zhang et al. [53]  Funded | RCT | Chinese residents with distress  China | 50.12  (6.61) | 25/26 | - Brief online mindfulness-based intervention, 2 hr. training/psycho-education session on mindfulness, group supported mindfulness practice | 0 | | BSI-8 |

Measure: GAD-7: General Anxiety Disorder-7; BAI: Beck Anxiety Inventory; HADS=The Hospital Anxiety and Depression Scale; DASS=Depression Anxiety Stress Scale; PHQ-4=Patient Health Questionnaire; STAI=The State-Trait Anxiety Inventory; PROMIS=Patient-Reported Outcomes Measurement Information; BSI-8=Brief Symptom

Inventory-8.
